# Vasohibin-2 modulates tumor onset in the gastrointestinal tract by normalizing tumor angiogenesis

**DOI:** 10.1186/1476-4598-13-99

**Published:** 2014-05-04

**Authors:** Shuji Kitahara, Yasuhiro Suzuki, Masae Morishima, Asuka Yoshii, Sachiko Kikuta, Kazuhiko Shimizu, Shunichi Morikawa, Yasufumi Sato, Taichi Ezaki

**Affiliations:** 1 Department of Anatomy and Developmental Biology, School of Medicine, Tokyo Women’s Medical University, 8-1 Kawada-cho, Shinjuku-ku, Tokyo 162-8666, Japan; 2 Department of Vascular Biology, Institute of Development, Aging and Cancer, Tohoku University, 4-1 Seiryo-machi, Aoba-ku, Sendai 980-8575, Japan

**Keywords:** Tumor vessel, Angiogenesis, *Apc*^*Min/+*^ mice, Vasohibin-2

## Abstract

**Background:**

Vasohibin-2 (VASH2) has been identified as an endogenous and vascular endothelial growth factor (VEGF)-independent angiogenic factor that is highly expressed in tumor cells. In the present study, we aimed to determine whether pre-existing vascular changes can be used to predict tumor transformation as benign or malignant. We sought to characterize microvascular changes and tumor development in the intestinal tract of *Apc*^*Min/+*^ mice and *Apc*^*Min/+*^/*Vash2*^*-/-*^ mice.

**Methods:**

*Apc*^*Min/+*^ mice provide a unique orthotopic model for the development of spontaneous adenomatous polyposis and subsequent carcinomas, a phenomenon termed the adenoma-carcinoma sequence. *Apc*^*Min/+*^ mice were mated with *Vash2*^*-/-*^ mice with a mixed C57BL/6 background and the resulting pups were screened for the Min mutation and for the *Vash2*^*-/-*^ gene by PCR. Intestinal tumors from *Apc*^*Min/+*^ mice and *Apc*^*Min/+*^/*Vash2*^*-/-*^ mice were removed and either frozen or epon-embedded for subsequent analyses. For 3-dimensional imaging using confocal laser-scanning microscopy and transmission electron microscopy, cryosections were made, and immunofluorescent staining for various markers was performed.

**Results:**

We found that structural abnormalities in tumor vessels from benign tumors resembled those in malignant tumors. In addition, a novel angiogenic factor, vasohibin-2 (VASH2) protein, was detected around tumor blood vessels in late-stage adenomas and adenocarcinomas, but was absent from early-stage adenomas in *Apc*^*Min/+*^ mice. Tumors used to examine endogenous VASH2 (derived from CMT93 colon carcinomas) were less vascularized in *Vash2*^-/-^ mice and were more regular than those seen in wild-type (WT) mice. In addition, tumors in *Vash2*^-/-^ mice were smaller than those in WT mice. Furthermore, cross-breeding of mice homozygous for a deletion of *Vash2* with mice heterozygous for the *APC* mutation resulted in animals that showed a significant decrease in the number of polyps in the small intestine.

**Conclusion:**

We propose that VASH2 may modulate the onset of tumors in the gastrointestinal tract by regulating tumor angiogenesis.

## Background

Angiogenesis, the formation of new blood vessels, is an essential physiological process in embryo development, normal growth, and tissue repair, and is tightly regulated at the molecular level. Dysregulation of angiogenesis occurs in various pathological conditions and is one of the hallmarks of cancer [1,2]. Recognition of the role of angiogenesis during neoplastic development is important for a more comprehensive understanding of the mechanisms involved in tumor growth and metastasis [3-5].

Tumor vessels are histopathologically different from normal vessels; most tumor vessels have irregular diameters, abnormal branching patterns, and do not fit well into the usual categorization of arterioles, capillaries, or venules [6-8]. Moreover, the endothelial cells (ECs) that make up tumor vessels are often loosely interconnected and have intercellular openings and abnormal pericytes, contributing to the leakiness of these vessels [9-11]. Structural abnormalities in the basement membrane of tumor vessels are also responsible for their relative immaturity in comparison with normal vessels [12,13].

Although the main purpose of tumor angiogenesis is to maintain blood supply to the tumor, the process usually occurs in an abnormally regulated fashion and the resulting tumor vasculature may have abnormal organization, structure, and function [1]. Recent advances have led to a better understanding of the vascular changes in malignant tumors, but the structural abnormalities in blood vessels of benign tumors (pre-neoplastic lesions) and those present during malignant transformation are still poorly understood.

Angiogenesis is regulated by highly coordinated functions of various proteins that play pro- or anti-angiogenic roles [14]. Pro-angiogenic factors include vascular endothelial growth factor (VEGF), fibroblast growth factor, platelet-derived growth factor, insulin-like growth factor, transforming growth factors, angiopoietins, and several chemokines, while anti-angiogenic factors include thrombospondin-1, angiostatin, and endostatin [15]. Two novel endogenous paracrine factors, termed vasohibins, have also been described recently [16]. Vasohibin-1 is anti-angiogenic, while vasohibin-2 appears to be pro-angiogenic. Vasohibin-2 (VASH2) is mainly expressed by infiltrating bone marrow-derived mononuclear cells at the angiogenic-sprouting front [16-20], and the expression of VASH2 in human serous ovarian adenocarcinoma and hepatocellular carcinoma accelerates tumor growth by promoting angiogenesis [21,22]. However, it is not known if there are differences in VASH2 expression between tumor cells and tumor-associated ECs. In addition, although VEGF-targeted therapy shows promise for the inhibition of angiogenesis during tumor progression, new therapeutic targets are needed to advance anti-angiogenic treatments in cancer. Therefore, the development of novel therapeutic agents may be facilitated by identification and characterization of new angiogenic factors, and this may be achieved using spontaneous tumor models [22].

Elucidation of the angiogenic patterns in benign tumors and the involvement of various pro- and anti-angiogenic factors are important for understanding the development of malignant tumors and the neoplastic transformation sequence in intestinal epithelia [23,24]. In the current study, relationships between tumor angiogenesis and multi-step carcinogenesis were assessed using the *Apc*^*Min/+*^ mouse model, which spontaneously develops multiple intestinal adenomas that mimic those that undergo early transformation into adenocarcinomas in patients with familial adenomatous polyposis [25]. Using this mouse model offers the advantage of close recapitulation of the histopathological characteristics observed in human cancer. Furthermore, tissue-specific induction of mutations gives rise to orthotopic tumors in the context of a functional, immune-competent microenvironment, and thus includes the crosstalk between an emerging tumor and its environment [26-28].

In the present study, we sought to characterize the microvascular changes that occur during the adenoma-carcinoma sequence in a tumor to determine whether pre-existing vascular changes can be used to predict tumor transformation from benign to malignant. For comparison, wild-type (WT) C57BL/6 mice and *Vash2*^*-/-*^ mice bearing transplanted syngeneic CMT93 colorectal carcinoma cells were included in the study. The effects of VASH2 on adenoma growth and progression to carcinomas were also examined by cross-breeding mice with a complete absence of *Vash2* expression (*Vash2*^*-/-*^ mice) with *Apc*^*Min/+*^ mice. Our results indicate a novel role for VASH2 in tumor angiogenesis as an index of malignant transformation and suggest that VASH2 may be a novel target for anti-angiogenic agents in cancer therapy.

## Results

### Functional changes in local vascular networks during the adenoma-carcinoma sequence

**Figure 1:**
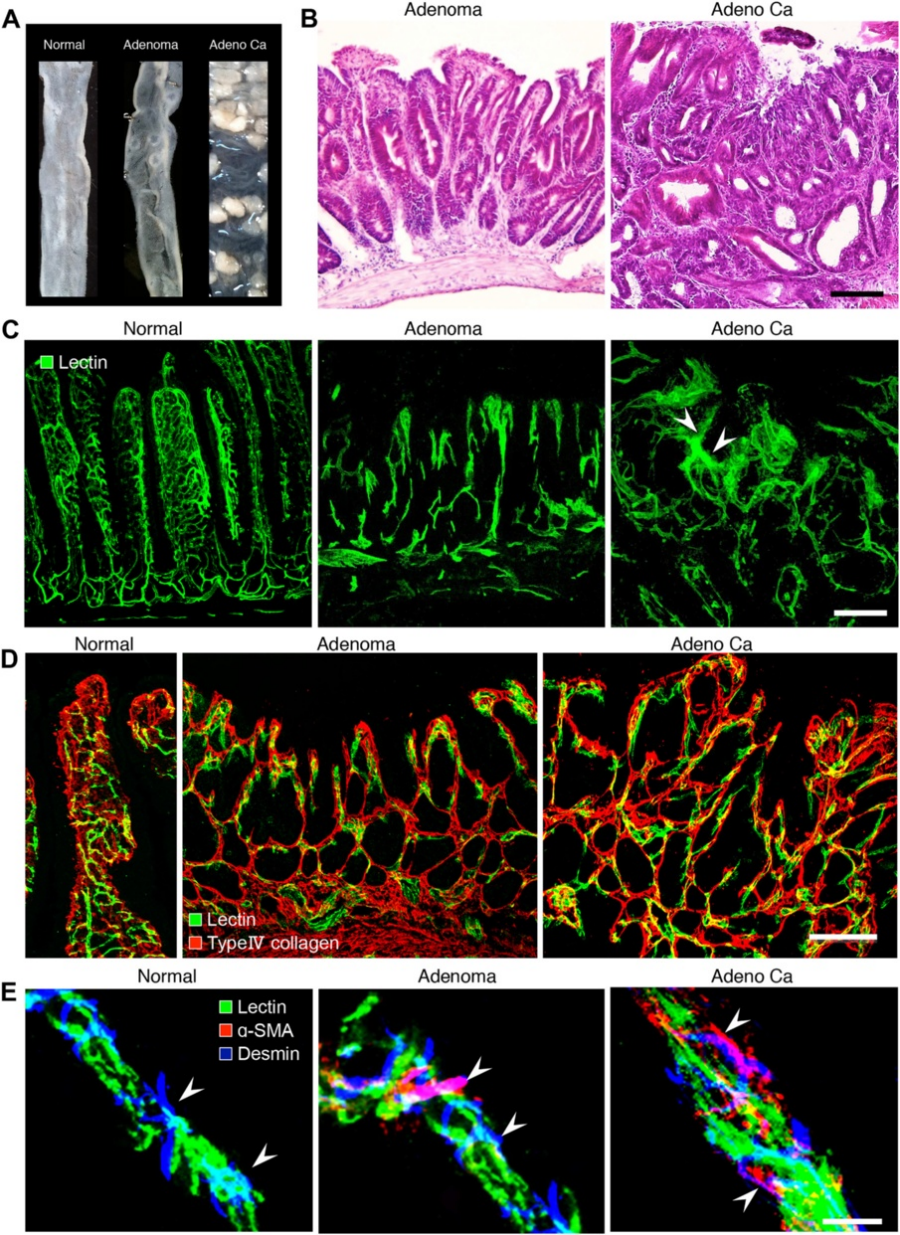
**Relationship between tumor progression and local microvascular changes during multistep carcinogenesis in *Apc***^***Min/+***^ **mice. A**, Representative images of normal intestine or intestinal polyps: normal region (left), adenoma (center), adenocarcinoma (right). n = 20. **B**, H&E staining of adenoma and adenocarcinoma. Scale bars: 100 μm. **C**, Tomato lectin-labeled vascular architecture in a normal region, adenoma, and adenocarcinoma. Note the marked structural abnormalities in the tumor vessels, including altered vascular density, vessel compression, uneven diameter, blind ending vessels, and leakiness (arrowheads) in adenocarcinoma. Blood vessels in adenoma and adenocarcinoma lost their vascular hierarchy. Scale bars: 100 μm. **D**, Double-staining for FITC-tomato lectin and type IV collagen in a normal region, adenoma and adenocarcinoma during the adenoma-carcinoma sequence. Scale bars: 100 μm. **E**, Pericyte distribution in tumor vessels during the adenoma-carcinoma sequence in *Apc*^*Min/+*^ mice. Vessels in a normal region of the small intestine, adenomas, and adenocarcinomas. Vessels were stained with FITC-tomato lectin (green). Pericytes were visualized with a combination of α-SMA (red) and desmin (blue). Scale bars: 20 μm.

To understand the process of neovascularization in solid spontaneous tumors, the relationship between tumor progression and tumor vessel formation during the adenoma-carcinoma sequence was examined using tomato lectin and immunohistochemistry (Figure 1). Adenoma and adenocarcinoma were recognizable macroscopically in the intestine (Figure 1A) and in hematoxylin and eosin (H&E)-stained specimens (Figure 1B). Abnormal tumor vessels formed in adenomas were highly dilated compared with normal intestinal vessels (Figure 1C). Additionally, abnormally shaped new tumor vessels appeared rapidly in the adenoma. Although the vascularized areas in the tumor became wider and the density of the tumor vessels increased as tumors grew and progressed towards malignancy, the growing tumor vessels also spread toward the outside of the tumors. Leakiness of tumor vessels was also seen in adenocarcinoma. In contrast, leakage of lectin from blood vessels was not observed in normal tissue or in adenoma (Figure 1C).

### Morphological changes of the vascular walls in adenoma and adenocarcinoma

To illustrate the histopathological basis of the changes observed during the sequence of malignant changes, basement membranes outlined by type IV collagen in benign intestinal tumors were compared with those in malignant intestinal tumors from *Apc*^*Min/+*^ mice. Lectin staining extended clearly beyond the basement membranes of the blood vessels in the tumors. As the tumors became malignant, the irregularity of the epithelial framework became more prominent (Figure 1D). Moreover, triple fluorescent staining for tomato lectin, desmin, and α-smooth muscle actin (α-SMA) in normal intestinal tissue, adenoma, and adenocarcinoma (Figure 1D) revealed that α-SMA-positive pericytes, which were absent in normal capillaries [9], were clearly present in both adenoma and adenocarcinoma samples. These results indicate that the blood vessels in pre-neoplastic lesions (benign tumors) already exhibit malignant patterns.

### Ultrastructure of newly developed tumor vessel endothelial cells in adenoma and adenocarcinoma

**Figure 2:**
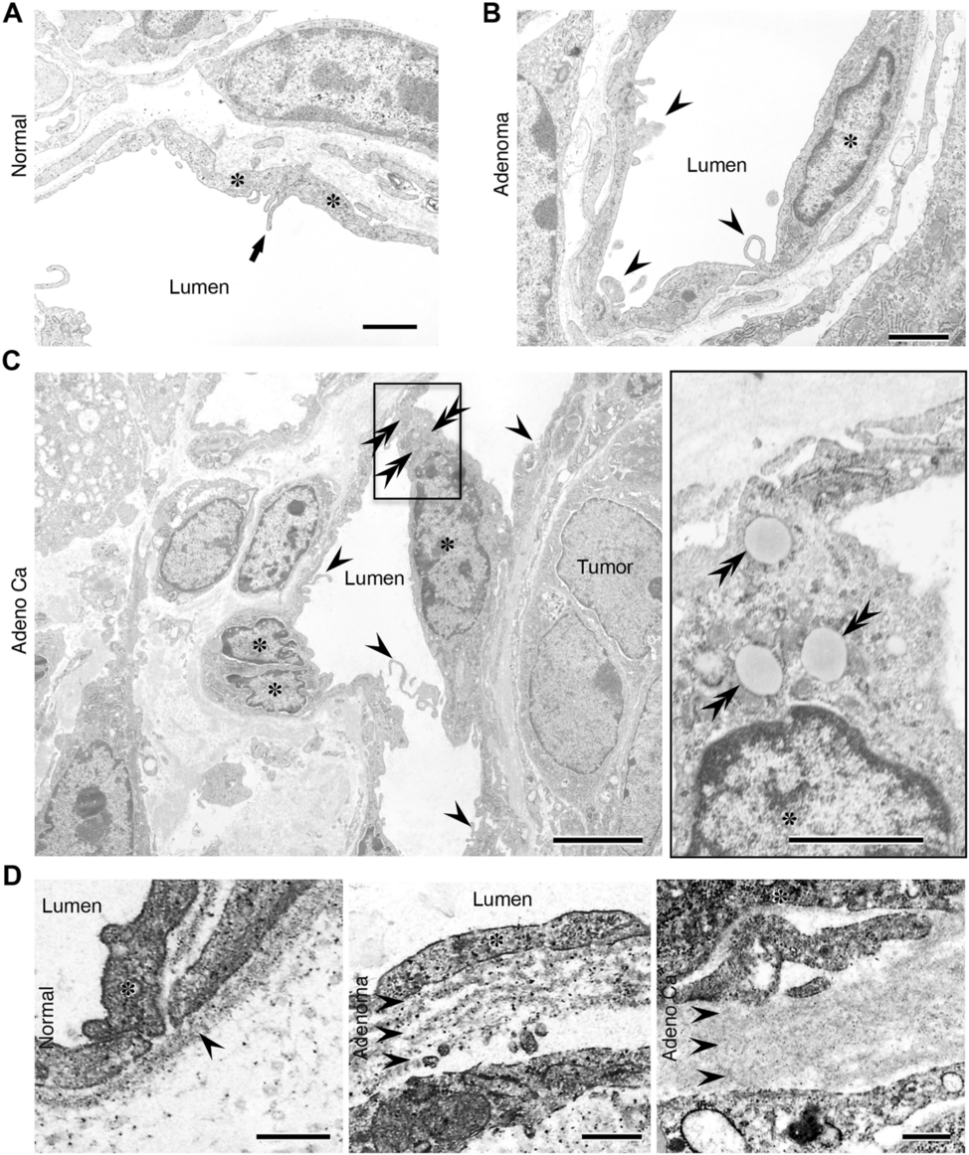
**Ultra-thin transmission electron microscope sections showing morphological changes in blood vessels during the adenoma-carcinoma sequence in *Apc***^***Min/+***^ **mice. A**, A vessel in a normal region. The luminal surface of the endothelium generally has a smooth contour, but the thin margins of adjacent cells may overlap slightly and the distance to the lumen is short. Asterisks, endothelial cells; arrows, marginal fold at the cellular edge. Scale bars: 2 μm. **B**, Adenoma. Marked structural changes in the microvasculature were observed in aberrant villi of benign tumors that lacked endothelial cell junctions. Asterisks, endothelial cells; arrowheads, abnormal microvilli. Scale bars: 2 μm. **C**, Adenocarcinoma. The morphology of the vascular luminal surface became irregular; the endothelial cells accumulated lipid droplets and the irregularity of tumor vessels in terms of size variation became more prominent. Asterisks, endothelial cells; arrowheads, abnormal microvilli; double arrowheads, lipid droplet. Scale bars: 4 μm (left), 1 μm (right). **D**, Ultra-thin sections showing morphological changes in the basement membrane. Note the multi-layered basement membranes in adenoma and adenocarcinoma. Asterisks, endothelial cells; double arrows, a layer of the basement membrane. Scale bars: 0.5 μm.

The ultrastructure of blood vessel ECs was examined during the adenoma-carcinoma sequence in *Apc*^*Min/+*^ mice. Compared with normal blood vessels (Figure 2A), abnormal microvilli and membrane projections were observed in adenoma and adenocarcinoma tumor blood vessels (Figure 2B and C). Marked structural changes in the microvasculature were found in the microvilli of benign tumors (Figure 2B). As the tumors became malignant, the irregularity of microvilli in the tumor endothelium became more prominent and more frequent (Figure 2C), and ECs accumulated lipid droplets in adenocarcinomas. At the same time, the basement membrane of tumor vessels became more multi-layered in adenoma and adenocarcinoma, compared with normal vessels (Figure 2D).

### Angiogenic features of local microvessels in adenoma and adenocarcinoma

**Figure 3:**
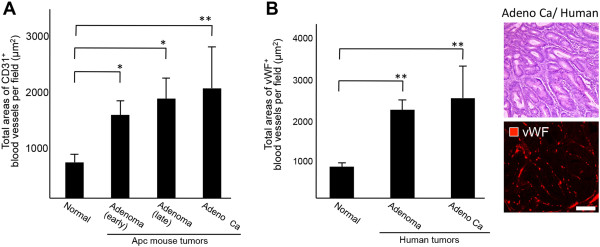
**Comparison of vascular density and angiogenic patterns between mouse intestinal polyps and human surgical specimens. A**, Vascular density and angiogenic patterns of normal intestine and tumor tissues in *Apc*^*Min/+*^ mice. The difference in density of microvessels per field between normal tissue and adenoma or adenocarcinoma in *Apc*^*Min/+*^ mice was compared by measuring the total area comprising vessels at each stage. The MVD was estimated by measuring the total areas of normal blood vessels and newly formed blood vessels in 10 separate fields of normal small intestine, mouse adenoma, or mouse adenocarcinoma at a magnification of 600 ×. ^*^P < 0.05, ^**^P < 0.01. **B**, Vascular density and angiogenic patterns of normal human intestine and colon tumor specimens. The MVD was estimated by measuring total areas comprising blood vessels in 10 separate fields of normal colon, human adenoma, or human adenocarcinoma at a magnification of 600 ×. Hematoxylin and eosin (H&E) staining of adenocarcinoma in human specimens and staining for vWF, a marker for human endothelial cells, in adenocarcinoma. ^**^P < 0.01. Scale bar: 100 μm.

Next, we compared differences in the densities of microvessels per field between normal tissue and adenoma or adenocarcinoma in *Apc*^*Min/+*^ mice by measuring the total area of the vessels at each stage (Figure 3A). The densities of CD31-positive vessels were significantly greater in adenoma and adenocarcinoma than in normal tissues in *Apc*^*Min/+*^ mice (Figure 3A). Moreover, the densities of Von Willebrand factor (vWF)-positive vessels were significantly greater in adenoma and adenocarcinoma than in normal tissues in human specimens (Figure 3B). These results show that the microvascular densities of human adenoma and adenocarcinoma were similar to those of *Apc*^*Min/+*^ mouse tumors (Figure 3).

Few studies have reported the mRNA expression of endothelial markers and angiogenic factors in benign tumors. We therefore performed real-time PCR analysis of normal intestinal tissues or intestinal polyps isolated from WT mice or *Apc*^*Min/+*^ mice (Additional file 1: Figure S1). These data showed significant increases in the expression of *CD31* and *CD105* in intestinal polyps from *Apc*^*Min/+*^ mice compared with normal intestinal tissues from WT and *Apc*^*Min/+*^ mice. The expression of *CD105* in intestinal polyps from *Apc*^*Min/+*^ mice was also significantly increased in comparison with that in WT mice. In contrast, no significant changes in the expression of *Vegfa* were observed among all the groups.

### Vash2 expression in tumor blood vessels and during malignant transformation

**Figure 4:**
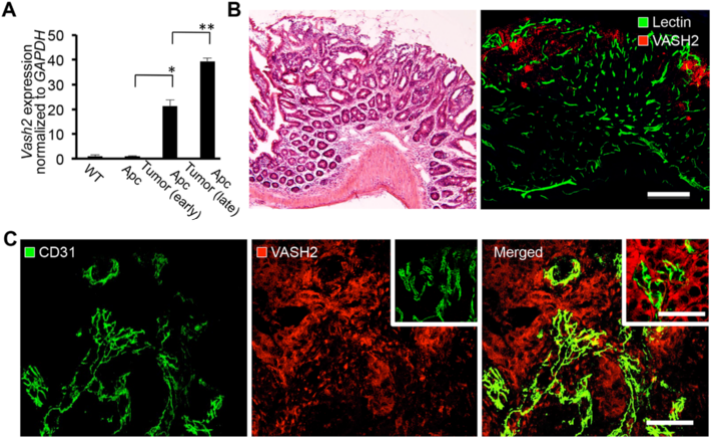
**Expression of VASH2 protein in tumors during the adenoma-carcinoma sequence. A**, Normal intestinal tissue or intestinal polyps were examined for *Vash2* expression by RT-PCR. Total RNA was isolated from normal intestinal tissue in C57BL/6 mice and *Apc*^*Min/+*^ mice or from intestinal polyps in *Apc*^*Min/+*^ mice. All samples were normalized to *Gapdh* and are expressed as relative ratios to wild-type (WT) controls. Note the increase in *Vash2* levels in late-stage adenoma and adenocarcinoma in *Apc*^*Min/+*^ mice. ^*^P < 0.05, ^**^P < 0.01, n = 3. **B**, H&E staining of late-stage adenoma in *Apc*^*Min/+*^ mice and double-immunostaining of tomato lectin and VASH2 for detection of tumor vessels in the same region. Scale bar: 200 μm. **C**, A high magnification image of CD31 staining in adenocarcinoma, showing VASH2 expression around tumor vessels. Note that VASH2 expression was only found around these vessels in adenocarcinoma. Center inset, control staining with a nonspecific IgG. Right inset, higher magnification of tumor cells shown in D. Scale bar: 50 μm, 30 μm (right inset).

**Figure 5:**
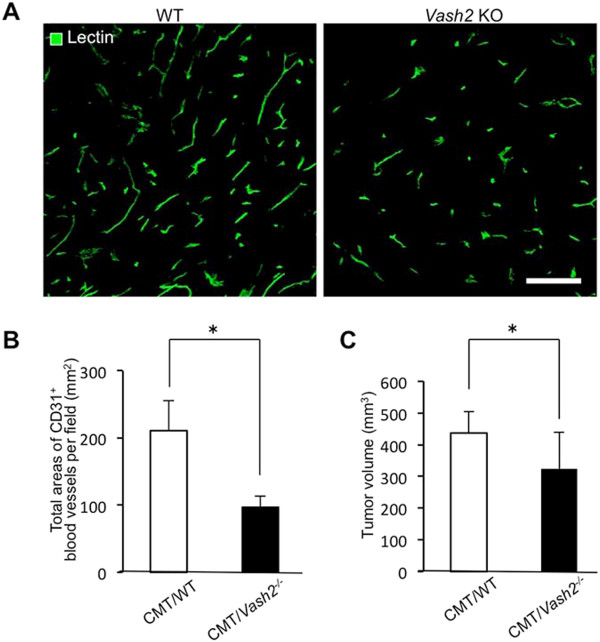
**Tumor progression and tumor angiogenesis in *Vash2 *KO mice. A**, Tomato lectin staining of tumor vessels in CMT93 tumor-bearing WT mice (left) and *Vash2*^-/-^ mice (right). Scale bars: 100 μm. Compared with CMT93 tumors in WT mice, the number of tumor vessels markedly decreased in tumors of *Vash2*^-/-^ mice. **B**, Microvascular density (MVD) was estimated by measuring the total area comprising CD31^+^ blood vessels in three separate fields of CMT93 tumors in WT mice or *Vash2*^-/-^ mice at 400× magnification. ^*^P < 0.05, n = 8. **C**, Growth of CMT93 tumors transplanted into WT or *Vash2*^-/-^ mice. Open columns represent CMT93 tumor-bearing WT mice and closed columns represent CMT93 tumor-bearing *Vash2*^-/-^ mice. ^*^P < 0.05, n = 15.

Unlike VEGF and other angiogenic factors, VASH2 is an endogenous and VEGF-independent angiogenic factor that is highly expressed in bone marrow-derived mononuclear cells and tumor cells, but only weakly expressed in ECs [19,21]. Its role in tumor angiogenesis is unknown, but it is likely that VASH2 functions via mechanisms that are distinct from those of VEGF [16]. Therefore, we examined the expression of VASH2 during the malignant transformation sequence in *Apc*^*Min/+*^ mice. The level of *Vash2* mRNA increased during malignant transformation (Figure 4A). Moreover, VASH2 protein was detected around tumor blood vessels in late-stage adenomas and adenocarcinomas, while early-stage adenomas stained negative for VASH2 in *Apc*^*Min/+*^ mice (Figure 4B and C). In addition, the expression of VASH2 was detected in tumor cells from adenocarcinomas (Figure 5C).

### Effects of Vash2-knockout on tumor growth and angiogenesis in C57BL/6 mice

VASH2 protein was detected around tumor blood vessels in late-stage adenoma and adenocarcinoma in *Apc*^*Min/+*^ mice. These findings suggested that the expression of VASH2 might contribute to malignant transformation during the adenoma-carcinoma sequence. Thus, to clarify the function of endogenous VASH2, we examined whether tumor growth and tumor angiogenesis were altered in *Vash2*^-/-^ mice. CMT93 tumors expressing VASH2 were implanted by injecting WT and *Vash2*^*-/-*^ C57BL/6 mice with 2.5 × 10^6^ cells (Additional file 2: Figure S2 and Figure 5). Tumors in *Vash2*^-/-^ mice were less vascularized and blood vessels appeared more normal than those in WT mice (Figure 5A and B). In addition, tumors in *Vash2*^-/-^ mice were smaller than those in WT mice (Figure 5C).

### VASH2 modulates tumor onset in the gastrointestinal tract of Apc^Min/+^/Vash2^-/-^ mice

**Figure 6:**
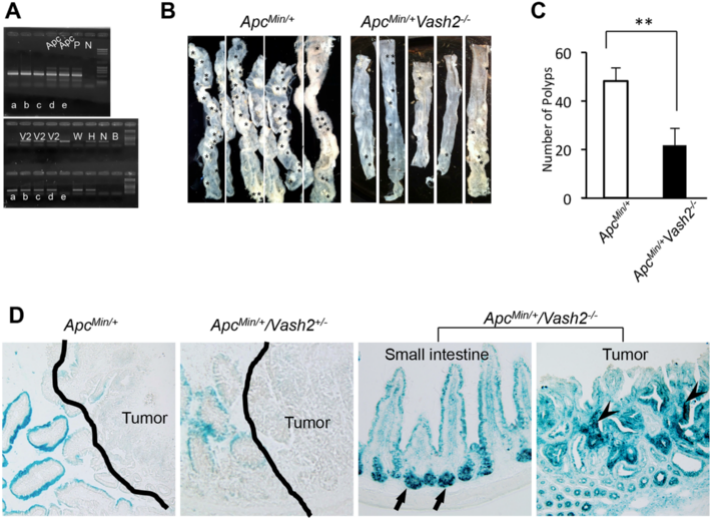
**Tumor onset in the intestinal tract of *Apc***^***Min/+***^**/*Vash2***^***-/-***^ **mice. A**, Genomic DNA samples isolated from mouse tails were genotyped by PCR using primers that detect both WT and mutant alleles of *Apc*^*Min/+*^ or *Vash2*. **B**, Dissection micrographs of the small intestine from *Apc*^*Min/+*^ mice (left) and *Apc*^*Min/+*^/*Vash2*^*-/-*^ mice (right) at 16 weeks of age. **C**, Polyp numbers in the small intestine in *Apc*^*Min/+*^ mice (open column) or *Apc*^*Min/+*^/*Vash2*^*-/-*^ mice (closed column) at 16 weeks of age. ^**^P < 0.01, n = 8. **D**, LacZ activity in *Apc*^*Min/+*^mice, *Apc*^*Min/+*^/*Vash2*^*+/-*^ mice and *Apc*^*Min/+*^/*Vash2*^*-/-*^ mice. Expression of LacZ was not seen in *Apc*^*Min/+*^ mice or in *Apc*^*Min/+*^/*Vash2*^*+/-*^ mice. Note the marked LacZ expression in crypts (arrows) and tumor cells in *Apc*^*Min/+*^/*Vash2*^*-/-*^ (arrowheads). Line, tumor area.

To investigate the possible roles of VASH2 in intestinal tract tumorigenesis, *Apc*^*Min/+*^ mice were crossed with *Vash2*^*-/-*^ mice to generate *Apc*^*Min/+*^/*Vash2*^*-/-*^ mice (Figure 6A). Polyp numbers were then compared between *Apc*^*Min/+*^ mice and *Apc*^*Min/+*^/*Vash2*^*-/-*^ mice (Figure 6B and C). The number of small intestinal polyps was significantly reduced in *Apc*^*Min/+*^/*Vash2*^*-/-*^ mice at 16 weeks of age compared with *Apc*^*Min/+*^ mice (Figure 6C). Histologically, all polyps in *Apc*^*Min/+*^/*Vash2*^*-/-*^ mice were adenomas or adenocarcinomas, similar to those in *Apc*^*Min/+*^ mice.

To determine the relationship between VASH2 suppression and intestinal polyposis, we assessed *Vash2* mRNA levels in the intestinal mucosa and polyps. Because we had inserted a β-galactosidase gene (*LacZ*) into the *Vash2* knockout allele, we monitored *Vash2* promoter activity by LacZ expression (Figure 6D). Strong X-gal staining was observed in the crypt of the normal small intestine and in tumor cells in adenoma or adenocarcinoma in *Apc*^*Min/+*^/*Vash2*^*-/-*^ mice. These results suggest that inhibition of VASH2 was reduced during polyposis in the small intestine of *Apc*^*Min/+*^ mice.

### VASH2 regulation of tumor blood vessels in polyps from Apc^Min/+^/Vash2^-/-^ mice

**Figure 7:**
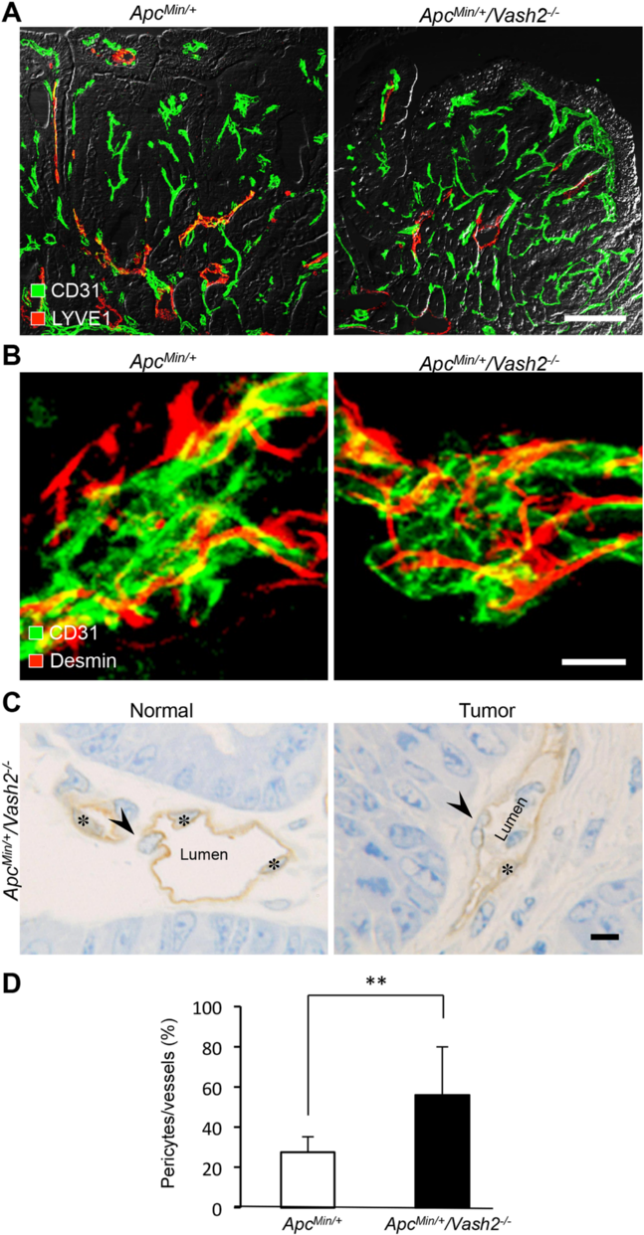
**Immunohistochemical analysis of tumor vessels in *Apc***^***Min/+***^ **and *Apc***^***Min/+***^**/*Vash2***^***-/-***^ **mice. A**, Double staining for CD31 and LYVE1 in intestinal polyps from *Apc*^*Min/+*^ mice and *Apc*^*Min/+*^/*Vash2*^*-/-*^ mice at 16 weeks of age. Scale bar: 100 μm. n = 3. **B**, Double staining for CD31 and Desmin of tumor vessels in intestinal polyps from *Apc*^*Min/+*^ mice and *Apc*^*Min/+*^/*Vash2*^*-/-*^ mice. Note the pericytes were detached from endothelia in *Apc*^*Min/+*^ mice, whereas they returned to cover vessel walls in *Apc*^*Min/+*^/*Vash2*^*-/-*^ mice. Scale bar: 10 μm, n = 3. **C**, Semi-thin section stained with toluidine blue and CD31. Pericyte coverage of normal tumor vessels was found in tumors of *Apc*^*Min/+*^/*Vash2*^*-/-*^ mice. Asterisks, endothelial cells. Arrowheads, pericytes. Scale bars: 10 μm, n = 3. **D**, Ratio of pericyte coverage. Open columns represent *Apc*^*Min/+*^ mice and closed columns represent *Apc*^*Min/+*^/*Vash2*^*-/-*^ mice. Pericytes per blood vessel was estimated by counting semi-thin section in 3 separate fields of tumor lesions in *Apc*^*Min/+*^ mice and *Apc*^*Min/+*^/*Vash2*^*-/-*^ mice at a magnification of × 1000. ^**^P < 0.01, n = 3.

**Figure 8:**
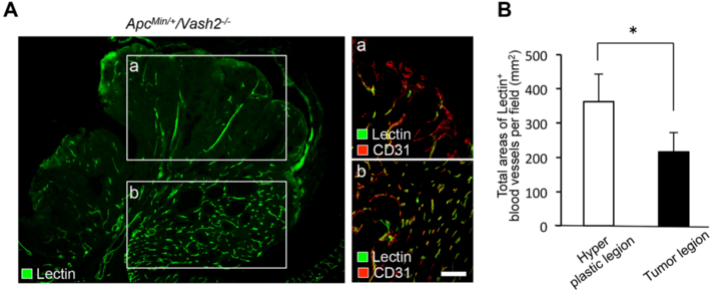
**Tomato lectin staining of functional tumor vessels in gastrointestinal polyps from *Apc***^***Min/+***^**/*Vash2***^***-/-***^ **mice. A**, Tomato lectin staining of functional tumor vessels in polyps. Double staining for tomato lectin and CD31 staining of tumor vessels in tumor lesions **(a)**. Double staining for tomato lectin and CD31 staining of tumor vessels in hyperplastic lesions **(b)**. Note the tumor lesions in *Apc*^*Min/+*^/*Vash2*^*-/-*^ mice were less vascularized than hyperplastic lesions in the gastrointestinal tract. Scale bars, 100 μm. **B**, MVD was estimated by measuring the total area of tomato lectin^+^ blood vessels in 5 separate fields of hyperplastic or tumor lesions in *Apc*^*Min/+*^/*Vash2*^*-/-*^ mice at a magnification of × 400. ^*^P < 0.05, n = 3.

To understand the role of VASH2 in tumor vessels, we compared the histopathological abnormalities of tumor vessels in *Apc*^*Min/+*^ mice and *Apc*^*Min/+*^/*Vash2*^*-/-*^ mice. Abnormal vascular networks appeared in tumors and were highly dilated compared with normal vessels (Figure 7A). However, tumor blood vessels in *Apc*^*Min/+*^/*Vash2*^*-/-*^ mice had more pericyte coverage compared with *Apc*^*Min/+*^ mice (Figure 7B-D). In contrast, some tumor lesions in *Apc*^*Min/+*^/*Vash2*^*-/-*^ mice were less vascularized than hyperplastic lesions in the polyps (Figure 8).

## Discussion

In this study, we sought to characterize microvascular changes in the intestine of *Apc*^*Min/+*^ mice, a useful animal model for studying spontaneous adenomatous polyposis and subsequent adenocarcinoma (a process termed as the adenoma-carcinoma sequence) [24,25]. We found that changes in local vascular networks reflected the neoplastic transformation sequence of the intestinal epithelia, as follows: First, both structural and functional changes in local vascular networks had already been initiated in benign tumors in *Apc*^*Min/+*^ mice, and in corresponding human surgical specimens. Second, the pattern of tumor angiogenesis in benign tumors was similar to that in malignant tumors. Third, VASH2 expressed in the adenocarcinoma cells promoted tumor growth and tumor angiogenesis in *Apc*^*Min/+*^ mice. Based on these data, we propose that the sequence of phases during tumor transformation from benign to malignant is based on microvascular changes, as summarized in Table 1.

**Table 1:** Changes in tumor microvasculature during multistep carcinogenesis

		**Apc mice**		
**Adenoma-carcinoma sequence:**	**Normal tissue**	**Adenoma**	**Adenoma**	**Adeno Ca**
		**(early)**	**(late)**	
Morphological abnormality;				
Vessel density	-	1+	2+	3+
Lumen	Round	Not round	Not round	Not round
	Systematic	Tortuous	Tortuous	Tortuous
Branching	-	1+	2+	3+
Irregularity	-	2+	3+	3+
Ec				
Morphology	Normal	Protuberance	Protuberance	Protuberance
		Microvilli	Microvilli	Microvilli
IHC Lectin	+	+	+	+
CD31	+	+	+	+
CD105	-	±	+	+
BM				
Morphology	Single	Multiple	Multiple	Multiple
Pc				
Morphology	Attached	Detached	Detached	Detached
		Protuberance	Protuberance	Protuberance
IHC Desmin	+	+	+	+
α-SMA	-	-	±	+
Pv				
IHC VASH2	-	-	+	+
Functional abnormality;				
Permeability	-	-	+	++
Blood flow (lectin)	Normal	Irregularity 1+	Irregularity 2+	Irregularity 2+
Hypoxia (HIF1-α)	-	-	±	+
Gene mutation	*Apc*	*Apc*	*K-ras*	*p53* and more
Stage of microvasculature	Stage 0	Stage I	Stage IIa	Stage IIb

*Apc*: *Apc*^*Min/+*^, *Ec*: Endothelial cell, *BM*: Basement membrane, *Pc*: Pericyte, *Pv*: Peripheral tumor vessel, *IHC*: Immunohistochemistry. Samples were scored as negative (-), mild (1+), Moderate (2+), Severe (3+). Positive staining was recorded as absent (-), weak (±), positive (+), strong (++).

Morphological changes in blood vessels occurred earlier than malignant changes in the epithelium of intestinal lesions. This suggests that angiogenesis patterns may play a critical role in the development and growth of benign tumors during multi-step carcinogenesis. There is increasing evidence that angiogenesis may also play a critical role in development of benign tumors [29-32]. Although angiogenesis is tightly regulated at the molecular level, dysregulation of angiogenesis is a hallmark of cancer and can lead to various pathological conditions [33]. The imbalance of pro- and anti-angiogenic signaling within tumors creates an abnormal vascular network that is characterized by dilated, tortuous, and hyperpermeable vessels [34]. The physiological consequences of these vascular abnormalities include temporal and spatial heterogeneity in tumor blood flow and oxygenation and increased interstitial fluid pressure in tumors [35,36]. These abnormalities and the resulting microenvironment fuel tumor progression and also lead to a reduction in the efficacy of chemotherapy, radiotherapy, and immunotherapies [37]. However, none of these previous studies have demonstrated that both structural and functional changes in blood vessels during multi-step carcinogenesis reflect the neoplastic transformation sequence of epithelia and the degree of malignancy, as we observed in this study. We were able to show changes in tumor vessels during multistep carcinogenesis in spontaneous tumors as a whole vascular network using fluorescent 3D imaging and transmission electron microscopy. These results showed that the histopathology of the vasculature in late stage adenoma was similar to that of malignant tumors.

VASH1 is expressed in ECs in the termination zone, suppressing angiogenesis, whereas VASH2 is expressed mainly in infiltrating bone marrow-derived mononuclear cells at the sprouting front, promoting angiogenesis [16,19]. However, exogenous VASH2 exhibits anti-angiogenic activity in the mouse cornea [17]. VASH2 expression has been demonstrated in certain ovarian cancers, where it promotes tumor growth and peritoneal dissemination of tumor cells by stimulating tumor angiogenesis [21]. VASH2 is also highly expressed in hepatocellular carcinoma cells (HCCs) and tissues, and promotes HCC angiogenesis and malignant transformation [22]. In our study, VASH2 was mainly expressed by late stage adenoma and spontaneous adenocarcinoma cells around tumor vessels in *Apc*^*Min/+*^ mice. Transplanted CMT93 tumors in *Vash2*^-/-^ mice were less vascularized and more regular than those in WT mice. Furthermore, in gastrointestinal tumors of *Apc*^*Min/+*^/*Vash2*^*-/-*^ mice, the number of small intestinal polyps was significantly reduced, pericyte coverage of tumor vessels was increased, and tumor lesions were less vascularized than hyperplasia lesions. These results support the hypothesis that VASH2 plays an important role in tumor angiogenesis and tumor progression. Because inhibition of VASH2 normalized abnormal tumor vessels in adenocarcinoma, VASH2 may be an important therapeutic target in the treatment of human cancers.

Control of angiogenic factors such as VASH2 at stages of benign tumorigenesis may inhibit malignant transformation of the epithelium. If new anti-vascular agents such as anti-VASH2 neutralizing antibodies could be developed to suppress changes in local vascular networks, the intestinal epithelium may not become malignant during the adenoma-carcinoma sequence. Thus, the findings of this study may contribute to the development of new antivascular agents as prophylactic medicines for malignant cancers. In addition, we propose that the microcirculation may act as an index of malignant transformation and may have potential use in future diagnosis and treatment of cancer. A better understanding of the various mechanisms of angiogenesis will facilitate development of novel anti-vascular therapies for the treatment of malignant tumors.

## Conclusions

We observed that angiogenic patterns were altered in pre-neoplastic intestinal lesions during the early stages of carcinogenesis, i.e., prior to the switch from adenoma to adenocarcinoma. The up-regulation of VASH2 in adenoma may induce the observed changes in vascular architecture, similar to those observed in malignant tumors. We conclude that several events such as "tumor angiogenesis" and the "malignant angiogenic switch" (summarized in Table 1) occur before transformation to carcinoma and are likely to reflect the continuous process of cancer development. We are currently investigating the possibility that this interaction also applies to other tumor models. Moreover, to provide a more comprehensive understanding of the angiogenic mechanisms of VASH2, further studies using *Apc*^*Min/+*^ / *Vash2*^*-/-*^ double-transgenic mice are underway. Further delineation of the role of VASH2 in tumor angiogenesis may lead to novel strategies in anti-tumor therapy.

## Methods

### Mice

C57BL/6 J-*Apc*^*Min/+*^ (*Apc*^*Min/+*^) of both sexes were purchased from the Jackson Laboratory (Bar Harbor, ME) and male C57BL/6 (wild type: WT, MHC class I type: H-2^b^) were purchased from Japan SLC Inc. (Shizuoka, Japan). *Vash2* knockout (*Vash2*^*-/-*^) mice were generated as described elsewhere [19]. *Apc*^*Min/+*^ mice of a pure C57BL/6 background were mated to *Vash2*^*-/-*^ mice of a mixed C57BL/6 background, and the resulting pups were screened for the Min mutation [38] and for the *Vash2*^*-/-*^ gene by PCR [19,39]. Mice were maintained in air-filtered clean rooms and fed sterilized standard laboratory chow and water *ad libitum*. Because it has been previously shown that a combination of a high-fat diet and dextran sodium sulfate strongly promotes intestinal carcinogenesis in *Apc*^*Min/+*^ mice [24,25,40], the animals were fed with a high-fat diet (Oriental Yeast, Tokyo, Japan) and 2% DSS (Tokyo Chemical Industry, Tokyo, Japan) to ensure development of adenomas and adenocarcinomas in the small intestine 3 months later. The Animal Experiment Committee, Tokyo Women’s Medical University (TWMU) approved the procedures employed in the handling and study of the mice. The following experiments were performed in accordance with legislation of the Institute of Laboratory Animals for Animal Experimentation at TWMU. Unless otherwise stated, at least 20 mice in each experimental group were examined.

### Cell culture

CMT93 cells [41] derived from a mouse rectal carcinoma were obtained from the European Collection of Cell Cultures (Sigma-Aldrich, St Louis, MO, USA). CMT93 cells were grown in Dulbecco’s modified Eagle medium (DMEM: Invitrogen, Carlsbad, CA, USA) supplemented with 10% heat-inactivated fetal bovine serum, 5 × 10^-5^ M 2-mercaptoethanol, 10 mM HEPES, 1 mM sodium pyruvate, 3.75 g/L NaHCO_3_, 2 mM glutamine, 100 U/mL penicillin, and 0.1 mg/mL streptomycin (Gibco, Grand Island, NY, USA). CMT93 tumor cells (2.5 × 10^6^ cells) in 250 μL of calcium- and magnesium-free phosphate-buffered saline (Ca^++^-, Mg^++^-free PBS; pH 7.4) were injected into the dorsal subcutis (s.c.) of WT C57BL/6 mice and *Vash2*^*-/-*^ mice.

### Definition of adenomas and adenocarcinomas

All tumors (early-stage: approximately 12 weeks or earlier, late-stage: approximately 16 weeks or later) that developed in mice were examined in paraffin sections stained with H&E, and histopathological changes such as carcinoma *in situ* and stromal invasion were evaluated. Tumors were diagnosed as adenomas by expansion to the mucosal layer, reduction of goblet cell numbers, and moderate loss of mucosal architecture by glandular growth and dilated cysts. Adenomas with 50% of high-grade dysplasia (severe distortion of the glandular architecture and prominent atypical cells) were considered carcinomas *in situ*. However, only the lesions showing invasion through the lamina muscularis mucosae were identified as adenocarcinomas.

### General tissue preparation

All mice were anesthetized by an intramuscular (i.m.) injection of ketamine (87 mg/kg) and xylazine (13 mg/kg). Under deep anesthesia, the chest was opened and the aorta was perfused with 4% paraformaldehyde (PFA) in PBS for 10 min at a pressure of 120 mm Hg using an 18-gauge cannula inserted via the left ventricle. The blood and fixative were then flushed out through an opening in the right atrium. After perfusion, tissues were removed, cut into small pieces and rinsed in PBS, then further immersed in PBS containing a graded series of sucrose (up to 30%) at 4°C overnight. Subsequently, these tissues were embedded in Tissue-Tek O.C.T. compound (Sakura Finetek, Torrance, CA, USA) and snap-frozen in liquid nitrogen. Cryostat sections (14–120 μm) were cut, placed on silane-coated glass slides, air-dried for at least 2 h and then immunostained.

To obtain semi-thin Epon-embedded sections, various tissues were excised, cut into small blocks, and fixed by immersion in 2% glutaraldehyde in 0.1 M phosphate buffer (PB; pH 7.2) at 4°C for 24 h. After washing out the fixatives with 0.1 M PB, the blocks were treated with 1% osmium tetroxide (O_S_O_4_)-0.1 M PB (a mixture of 2% OsO_4_ + 0.2 M PB). The tissues were dehydrated in a graded series of ethanol, infiltrated with propylene oxide, and embedded in Epon. Semi-thin sections (0.5 μm thick) were made and stained with 1% toluidine blue in PBS.

### Labeling of blood vessels with tomato lectin for 3d imaging

To identify blood vessels, we used the intravascular perfusion of fluorescent tomato lectin to label all blood-circulating vessels [42]. Briefly, under anesthesia, the mice were intravenously (i.v.) injected with 100 μl of FITC-conjugated tomato lectin (*Lycopersicon esculentum* lectin; 1 mg/mL; Vector Labs, Burlingame, CA). Tomato lectin binds uniformly to the luminal surface of ECs [43] and can be used to label all blood vessels that have a patent blood supply. After perfusion, the tissues were processed for subsequent analyses as described above.

### Immunohistochemistry

Cryosections were first incubated in 4% Block Ace (Dainippon Seiyaku, Osaka, Japan) to block nonspecific background stains, and successively incubated with various primary antibodies (alone or in combination) in PBS containing 1% bovine serum albumin (Sigma-Aldrich, St Louis, MO, USA) at 4°C overnight. ECs were identified with antibodies to CD31 (PECAM-1; hamster monoclonal, 1:400; Chemicon, Billerica, MA, USA) and Von Willebrand Factor (vWF; rabbit polyclonal antibody; dilution, 1:100; DakoCytomation, Glostrup, Denmark). The basement membrane was identified with an antibody against mouse type IV collagen (rabbit polyclonal 1:1000; Cosmo Bio, Tokyo, Japan). Pericytes were identified with antibodies to α-smooth muscle actin (Cy3-conjugated mouse monoclonal, 1:500; Sigma-Aldrich) and desmin (rabbit polyclonal, 1:200, Abcam, Cambridge, MA, USA). VASH2-expressing cells were labeled with a rabbit polyclonal antibody to mouse VASH2 [19] (1:100; a generous gift from the Institute of Development, Aging and Cancer, Tohoku University, Sendai, Japan). After several washes with PBS, specimens were incubated with combinations of fluorescent (FITC, Cy3, and Cy5)-conjugated anti-rat, -hamster, and -rabbit secondary antibodies (Jackson ImmunoResearch, West Grove, PA, USA) for 2 h at room temperature. Immunostained sections were examined using a Leica TCS-SL confocal laser-scanning microscope (Leica Microsystems, Wetzlar, Germany).

### Immunolabeling of semi-thin sections

After thawing and air-drying, cryosections were rehydrated in PBS then incubated with 4% Block Ace blocking solution (Dainippon Seiyaku, Tokyo, Japan) to reduce nonspecific background staining. For ordinary immunoenzymatic staining, tissue sections were incubated with anti-CD31 overnight at 4°C. The sections were further incubated with goat anti-rat immunoglobulins labeled with horseradish peroxidase (HRP) (GE Healthcare UK, Buckinghamshire, UK; 1:100 in PBS with 1% heat-inactivated normal mouse serum) for 2 h. The HRP reaction was developed at RT for 20 min in a solution of 10 mg of 3′-diaminobenzidine hydrochloride (DAB: Dojin Chemicals, Kumamoto, Japan) in 30 ml of PBS with 10 μg of 30% H_2_O_2_. Sections were washed in distilled water. After the DAB reaction, the sections were fixed in 2.5% glutaraldehyde in PB at 4°C for 1 h and subsequently in 2% osmium tetroxide in PB at room temperature for 1 h. They were then dehydrated in a graded series of ethanol and embedded in an epoxy resin. Semi-thin sections stained with 0.05% toluidine blue were examined using a light microscope.

### Morphometric analysis

For analyses of the microvessel density (MVD), the total areas of CD31, vWF-positive capillaries, and venules were assessed by scanning tumor sections under × 40 magnification and counting in 10 random fields under × 600 magnification [44,45]. Pericytes were identified by scanning tumor sections under × 1000 magnification and counting in 3 random fields under × 1000 magnification [46]. These data were analyzed using a BZ-Analyzer (Keyence, Osaka, Japan).

### Human tissue samples

Surgical specimens were obtained from 10 patients with colorectal adenoma (n = 5) or adenocarcinoma (n = 5) who underwent proctocolectomies in the Department of Surgery at Nishiarai Hospital (Tokyo, Japan) between April 2010 and March 2011. Patients with additional cancers were excluded. The Clinical Pathology Department of the Nishiarai hospital confirmed the histopathological diagnosis. Written informed consent was obtained from all patients for the surgery and for the use of their resected samples. H&E staining was performed to determine the histologic tumor type, lymphatic invasion, and vascular invasion in all specimens.

### Western blot analysis

Tissue and cell samples were lysed in SDS sample buffer, separated in 10% SDS-acrylamide gels, and electrotransferred to nitrocellulose membranes. After blocking with 5% non-fat dry milk in TBST buffer (10 mmol/L Tris–HCl (pH 8.0), 150 mmol/L NaCl, 0.05% Tween 20), the nitrocellulose membranes were probed with anti-CD31 (1:200; Santa Cruz Biotechnology, Santa Cruz, CA, USA), anti-VEGF (1:1,000; R&D Systems, MN, USA), anti-VEGF (1:1,000; R&D Systems, MN, USA), anti-CEA (1:2,000; Santa Cruz Biotechnology), anti-CA19-9 (1:2,000; Santa Cruz Biotechnology), anti-KRAS (1:2,000; Abcam, Cambridge, MA, USA), anti-p53 (1:1,000; R&D Systems), and anti-α-tubulin (1:1,000; Santa Cruz Biotechnology) antibodies, followed by incubation with HRP-conjugated anti-rabbit or anti-rat immunoglobulin G secondary antibodies (1:2,000; Jackson ImmunoResearch). The antibody binding was then visualized with enhanced chemiluminescence reagents (GE Healthcare, Amersham, UK), and the band images detected using the LAS3000 system (Fuji Film, Tokyo, Japan) were densitometrically analyzed using Image Gauge (Fuji Film).

### Quantitative real-time PCR

Total RNA was extracted using QIAzol Lysis Reagent (Qiagen, Venlo, Netherlands). First-strand cDNA was generated using ReverTra Ace (Toyobo, Osaka, Japan). Quantitative real-time RT-PCR was performed using the CFX96 real-time PCR detection system (Bio-Rad Laboratories, Hercules, CA, USA) according to the manufacturer’s instructions. PCR conditions consisted of an initial denaturation step at 95°C for 3 min, followed by 40 cycles of 10 s at 95°C, 10 s at 56°C, and 30 s at 72°C. Relative mRNA levels of target genes were normalized to the beta-2-microglobulin (*B2m*) mRNA level. The primer pairs used were as follows: mouse *B2m* forward, 5′-GGTCTTTCTGGTGCTTGTCTCA-3′, and reverse, 5′-GTTCGGCTTCCCATTCTCC-3′; mouse *CD31* forward, 5′-TTCAGCGAGATCCTGAGGGTC-3′, and reverse, 5′-CGCTTGGGTGTCATTCACGAC-3′; mouse *CD105* forward, 5′-TACAGTGCATCGACATGGAC-3′, and reverse, 5′- TCAGAGGTCAATGGAGACAC-3′; mouse *Vegfa* forward, 5′-AGAGAGCAACATCACCATGC-3′, and reverse, 5′- TCTGAACAAGGCTCACAGTG-3′; mouse *Vash2* forward, 5′-GGACATGCGGATGAAGATCT-3′, and reverse, 5′- CTAGATCCGGATCTGATAGC-3′.

### X-gal staining

Frozen sections were incubated in the dark for 18 h at 37°C in X-gal solution containing 1 mg/mL 5-bromo-4chloro-3-indolyl-β-d-galactoside (X-Gal, Gene Therapy Research Reagents, San Diego, CA, USA).

### Transmission electron microscopy

Anesthetized mice were fixed by vascular perfusion of 4% PFA and 2.5% glutaraldehyde in 0.1 M sodium cacodylate buffer (100 mL; pH 7.4) at a pressure of 120 mmHg. Immediately after the perfusion, the tumor tissues were removed, cut into small pieces, and immersed in the same fixative for another 2 h at 4°C. Specimens were then treated with 1% O_S_O_4_ for 2 h at 4°C, and then with saturated uranyl acetate for 3 h at room temperature. Thereafter, specimens were dehydrated in a graded series of ethanol and embedded in epoxy resin. Ultrathin sections (70 nm thick) were made, counterstained with saturated uranyl acetate followed by lead citrate, and observed using a Hitachi H-7000 electron microscope (Hitachi High-Technologies Co., Tokyo, Japan).

### Statistics

All results are expressed as mean ± standard deviation (SD). The statistical significance of differences was determined using the one-tailed Student’s *t*-test. The difference between two values was considered statistically significant if the *P* value was less than 0.05, and as highly significant if the *P* value was less than 0.01.

## Abbreviations

α-SMA: α-smooth muscle actin; ECs: Endothelial cells; HCCs: Hepatocellular carcinoma cells; vWF: Von Willebrand Factor; VASH2: Vasohibin-2; VEGF: Vascular endothelial growth factor.

## Competing interests

The authors declare that they have no competing interests.

## Authors’ contributions

SK and YS participated in the study design, performing the experiments, data analysis and drafting of the manuscript. KS and SM assisted with the in vitro, in vivo and cloning experiments and provided technical assistance. MM and SK provided material. AY performed immunohistochemistry experiments. YS was involved in the conception of the study and drafting the manuscript. TE participated in the study design, data analysis and writing of the manuscript. All authors read and approved the manuscript.

## Supplementary Material

Additional file 1: Figure S1 Expression of endothelial makers and angiogenic factors in normal intestinal tissue and intestinal polyp tissue as determined by quantitative RT-PCR. Total RNA was isolated and examined for *CD31*, *CD105*, and *Vegfa* expression. Data for all samples were normalized to *Gapdh* and expressed as relative ratios to wild-type (WT) controls. Note the increase in *CD31* and *CD105* levels in benign tumors in *Apc*^*Min/+*^ mice. WT, normal small intestine in C57BL/6 mice (white columns); Apc, normal small intestine in *Apc*^*Min/+*^ mice (slashed columns); Apc tumor 12 w, early stage (around 12 weeks) adenoma in *Apc*^*Min/+*^ mice (gray columns); Apc tumor 20 w, late stage (later than 20 weeks) adenoma or adenocarcinoma (black columns). ^*^P < 0.05, n = 3. Click here for file

Additional file 2: Figure S2 Expression of *Vash2* in CMT93 tumor cells as determined by quantitative RT-PCR. Total RNA was isolated and *Vash2* expression assessed. Data for all samples were normalized to *Gapdh* and are expressed as ratios relative to wild-type (WT) controls. n = 3. Click here for file
