# Supplementary figures and images for: Vasohibin-2 modulates tumor onset in the gastrointestinal tract by normalizing tumor angiogenesis
Source: Mol Cancer. 2014 May 4;13:99. doi: 10.1186/1476-4598-13-99 (PMC4113181; doi:10.1186/1476-4598-13-99)

Figure S1

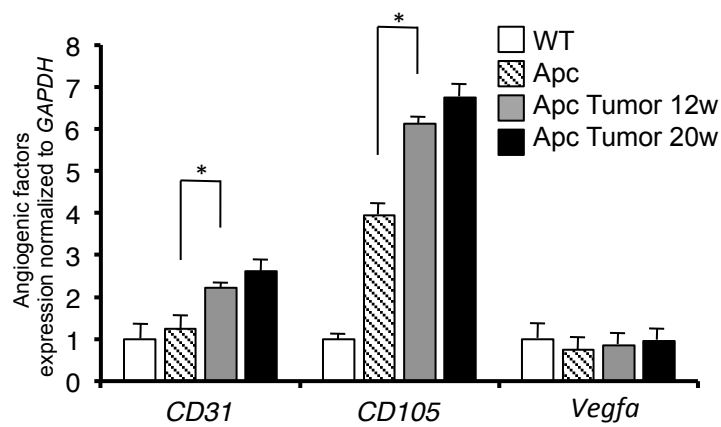

Supplement: Additional file 1: Figure S1 — Expression of endothelial makers and angiogenic factors in normal intestinal tissue and intestinal polyp tissue as determined by quantitative RT-PCR. Total RNA was isolated and examined for CD31, CD105, and Vegfa expression. Data for all samples were normalized to Gapdh and expressed as relative ratios to wild-type (WT) controls. Note the increase in CD31 and CD105 levels in benign tumors in Apc Min/+ mice. WT, normal small intestine in C57BL/6 mice (white columns); Apc, normal small intestine in Apc Min/+ mice (slashed columns); Apc tumor 12 w, early stage (around 12 weeks) adenoma in Apc Min/+ mice (gray columns); Apc tumor 20 w, late stage (later than 20 weeks) adenoma or adenocarcinoma (black columns). *P < 0.05, n = 3. [file 1476-4598-13-99-S1.pdf]

Figure S2

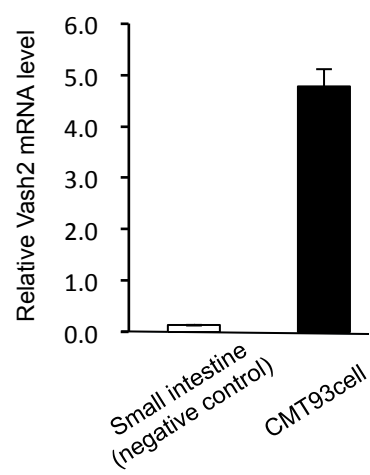

Supplement: Additional file 2: Figure S2 — Expression of Vash2 in CMT93 tumor cells as determined by quantitative RT-PCR. Total RNA was isolated and Vash2 expression assessed. Data for all samples were normalized to Gapdh and are expressed as ratios relative to wild-type (WT) controls. n = 3. [file 1476-4598-13-99-S2.pdf]
